# ‘But who’s gonna listen?’ A qualitative study of voicing experiences of healthcare in provincial correctional facilities in Ontario, Canada

**DOI:** 10.1017/S1463423626101340

**Published:** 2026-06-29

**Authors:** Katherine E. McLeod, Lindsay Jennings, George Flowers, James Ruston, Fiona G. Kouyoumdjian

**Affiliations:** Department of Family Medicine, McMaster University, Hamilton, Canada

**Keywords:** health, healthcare, lived experience, prisoners, prisons

## Abstract

Experiences of healthcare services are an important indicator of quality and health system improvement. In correctional facilities, structural and contextual factors affect experiences of healthcare services and processes for voicing these experiences. This study explores barriers and opportunities for people in custody to voice their experiences of healthcare services in custody. We held four focus groups and one interview with people living in the community who had accessed, or tried to access, healthcare services while incarcerated in a provincial correctional facility in Ontario, Canada in the previous five years. Using template analysis, we developed four interacting themes related to expectations and experiences of healthcare, and of submitting complaints or asking for help: i) the system is not designed for healthcare, ii) gatekeeping and perceptions of ‘deserving’ healthcare, iii) impact of healthcare on other outcomes, and iv) calling the abyss. These factors affected perceptions of the potential efficacy of a patient feedback process, and how people in custody were likely to engage with it. Participants also identified five key features that should be components of any patient feedback processes. This study highlights challenges to patient-reported experiences of care in quality improvement work in restrictive environments and with incarcerated populations.

## Introduction

Person-centred measures of healthcare, including patient experience and patient satisfaction (Larson *et al.*, 2019) reflect key dimensions of quality and are important to inform improvements in health services and systems (Doyle *et al*., 2013; Nundy *et al*., 2022). Assessments of experiences of healthcare are increasingly used in community healthcare settings (Bull *et al*., 2019) but are less common for healthcare provided in custody. In correctional facilities healthcare services are shaped by institutional policies and systems which prioritize security and risk management and may be in tension with patient-centred care (Sullivan *et al*., 2016). The carceral environment may also create unique opportunities and risks associated with providing feedback or participating in person-centred assessments of healthcare services (Hankins *et al.*, 2022b). Meaningful quality improvement informed by the experiences of people in custody requires understanding the barriers, risks, and opportunities related to voicing experiences of healthcare in these settings.

Healthcare services delivered in custody are framed by the structures, practices, and environment of correctional facilities. Common examples include policies limiting access to treatments or medications (Sullivan *et al*., 2016), limitations on the autonomy of healthcare providers who may report to correctional authorities (Pont *et al*., 2018), the role of correctional officers in facilitating or gatekeeping access to care (Condon *et al*., 2007; Plugge *et al*., 2008), and a lack of confidentiality (Condon *et al.*, 2007; Plugge *et al.*, 2008). These constraints may affect patient expectations of healthcare services (Plugge *et al*., 2008) and trust in quality improvement work. In addition, the environment of correctional facilities creates unique challenges to developing mechanisms for experiences of healthcare services to inform quality improvement. For example, correctional facilities are closed systems in which people in custody are dependent on facility staff and people may fear repercussions in expressing dissatisfaction with staff or services (van der Valk and Rogan, 2023). Conditions of confinement may also limit methods for appropriately collecting healthcare experience data including considerations of consent, confidentiality, and accessibility.

There has been limited research around experiences of healthcare services in correctional facilities (Hankins *et al.*, 2022a). Many studies that are available have focused on experiences of a specific service or sub-population, such as use of telemedicine (Jimenez-Galan *et al*., 2019; Colombo *et al*., 2022) or experiences of pregnant women (Alirezaei and Latifnejad Roudsari, 2022). Less is available about the idea of person-centred measures of care in custody. One scoping review examined how patient satisfaction with mental health services in correctional facilities has been measured (Jones *et al*., 2025), and one study examined the processes through which people could provide feedback or share experiences of the healthcare they received in two English prisons (Hankins *et al.*, 2022b).

The aim of this study was to explore facets of the correctional environment that affect experiences of healthcare services and to identify potential opportunities and barriers for people to voice their experiences or provide feedback about the healthcare that they receive in provincial correctional facilities in Ontario.

## Methods

Our research team consisted of a researcher (postdoctoral fellow, KM), a community researcher and activist with lived experience of incarceration (LJ), and a physician-researcher with experience providing care in correctional facilities (FK). Two community members with recent experience of incarceration also engaged with the research team as Advisors (GF, JR). In initial meetings Advisors provided guidance and feedback for the development of the project including on focus group questions, recruitment methods, and materials. After the analysis, early themes and interpretations were presented to Advisors for feedback and discussion.

To invite focus group participants, information about this study was distributed through email and social media via formal and informal networks. People 18 years or older who had experienced incarceration in a provincial correctional facility within the previous five years were invited to contact the research team to learn more about participating in the study. In Canada, people in provincial/territorial correctional facilities are people being held on remand (awaiting trial or sentencing) and people sentenced to less than two years in custody. Each potential participant was sent the consent information by email, then spoke with KM by phone to the review the consent document, provide consent, and answer demographic questions. Focus groups were held using remote video-conferencing software. To support accessibility of participation, potential participants were also offered one-on-one interviews if they were unable to join a focus group. The focus group/interview discussion guide is provided in Appendix A. All participants received an honorarium immediately following their focus group or interview.

Focus groups were audio-recorded and transcribed verbatim with identifying information removed. Each participant was randomly assigned a two-letter identifier. We used template analysis (King, 2012) to conduct a thematic analysis (Braun and Clarke, 2022). Template analysis is classified by Braun and Clark as a type of ‘codebook thematic analysis’ and is well suited to applied research because its structure lends efficiency while maintaining flexibility (King, 2012; Braun and Clarke, 2022). We created a set of a priori themes from the focus group discussion guide, existing literature on patient’s experiences and feedback on healthcare in custody, and our reading of the transcripts for familiarization with the data. Using these themes, one author (KM) created an initial codebook based on preliminary coding of one transcript. This codebook was reviewed by a second author (LJ) who co-facilitated three of the four focus groups and had read all transcripts. Once an initial codebook was agreed upon, (KM) applied the codebook to the remaining transcripts, revising the codebook as necessary. Finally, the first transcript was re-coded using the final codebook as a check of validity. Detailed record of the development of the template, including familiarization notes and all versions of the codebook, was kept as an audit trail (King, 2012; Braun and Clarke, 2022). Themes developed from the analysis were reviewed and discussed by all team members, including Advisors, in iterative meetings to establish final themes.

This study was approved by the Hamilton Integrated Research Ethics Board (Project # 15839).

## Results

We held four focus groups and conducted one interview in July and August 2023. Focus groups were between 68 and 83 minutes, the interview was 59 minutes long. In total, 13 people participated in these discussions, eight men, three women, and two people who identified as nonbinary. During the initial phone call each participant was asked to describe their race and/or ethnicity; one person identified as Indigenous, one person identified as Afro-Indigenous, five people identified as Black, African Canadian, or African Caribbean and six identified as White.

**Figure 1.**
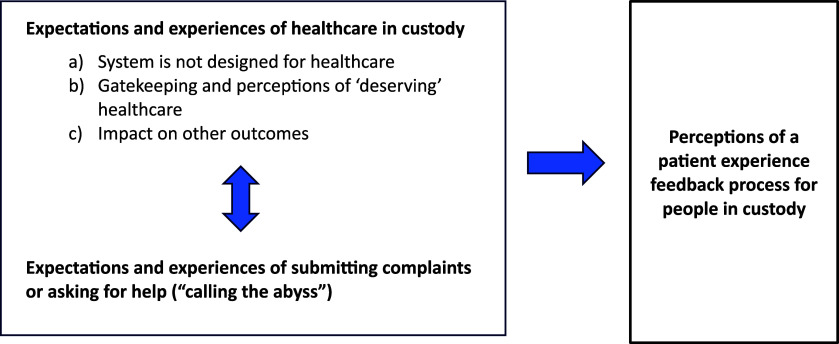
Experiences in custody identified by people with recent incarceration experience as affecting perceptions of, and engagement in, potential patient experience feedback processes.

Four interacting and interwoven themes highlighted factors related to expectations and experiences of healthcare in custody and of submitting complaints or asking for help that affect perceptions of patient experience feedback processes for addressing issues and quality improvement (Figure 1): 1) the system is not designed for healthcare, 2) gatekeeping and perceptions of ‘deserving’ healthcare, 3) impact of healthcare on other outcomes, and 4) ‘calling the abyss’. Participants also identified key features for processes aiming to include experiences of people in custody in work to improve healthcare services in correctional facilities.

### Expectations and experiences of healthcare in custody

#### System is not designed for healthcare

Participants emphasized that the purpose of the correctional system is to hold people in custody as a function of the legal system, not to provide healthcare. Participants saw that healthcare was a secondary priority for correctional facilities after security and control. An example given was that while in community you could choose who is in the room during your appointment, but in custody a correctional officer is present to hear you describe your health conditions to a nurse or doctor. Participants also frequently cited this hierarchy in discussions of access to medications. Medications were described as nearly impossible to get if you did not have an existing active prescription from a community physician. Medication could also be reduced or stopped all together to prevent hoarding or redistribution. Participants discussed that unlike in the community, people sometimes did not have a choice in refusing to take medication, as refusal could lead to adverse consequences.

*Like we’re at the mercy of a lot of other people a lot of the time… so it’s not like in regular society, where you have all this agency and autonomy to be able to take care of yourself. It’s almost like being a little kid again (ST).*

Participants described another feature of healthcare in custody is that it is designed to address acute healthcare needs while people are incarcerated, rather than long-term health and wellbeing.

*It seems like everything is done as a Band-aid or a stop gap. But there’s never a solution to the problem. It’s like a temporary little fix for right now, and that’s it (UA).*

This narrow view of the scope and aim of healthcare services in custody meant that people did not expect healthcare services in custody to consider or address health the same way they might in the community. This in turn was reflected in a lack of trust or expectation that there was appetite or opportunity for feedback of patient experiences to contribute to long-term quality improvement.

#### Gatekeeping and perceptions of ‘deserving’ healthcare

Participants described access to healthcare as being dependent on someone else’s assessment that you are ‘deserving’ of care. In one sense, people described difficulties accessing care for issues not seen as an emergency.

*A lot of time they don’t take you seriously unless you’re bleeding and not breathing. Then they worry about it. But if you’re breathing and can speak, ‘oh, you can wait’ seems to be the attitude (LC).*

The idea of deserving care was raised both in terms of healthcare staff’s prioritization of health issues to address, as well as the attitudes of correctional officers who facilitate access to healthcare. For example, correctional officers submit requests for healthcare completed by people in custody and escort people to medical appointments. The other way that participants talked about ‘deserving’ healthcare was through perceptions that access to care was tied to behaviour.

*Like they feel like you’ve gotten this privilege you don’t deserve, because you’re going to get somebody to look at your sores, or give you your pain meds, or give you your puffer… It’s not a privilege, but it feels like it’s this thing that you have to work towards and behave to receive (ST).*

This dynamic highlighted key challenges for feedback processes to be able to change power dynamics, that participants saw as inherent to the culture of corrections and staff.

#### Impact of healthcare on other outcomes

Throughout discussions, participants raised examples of ways that healthcare in custody is a determinant of other outcomes which affected both expectations and experiences of care. One poignant example was that not having access to medications, particularly for mental health conditions, could have a direct effect on legal proceedings and how a person is able to present or defend themselves in court.

*I actually refused to go to court in the morning…I said, I haven’t got my medication … I said, listen, you cannot place me in front of the courts in a manner that is not me in 100% state of mind. You can’t do that (UA).*

Participants also described fear that engaging with healthcare, or in complaints processes may be perceived negatively and could affect interactions with other systems such as child welfare agencies. One participant described seeing this fear affect new moms in custody.

*They don’t want to say anything, because they are already scared that their kid’s going be taken away from them by [child protective services] permanently for being in custody…so they’re suffering in silence because their goal is to get their kid back (BN).*

The idea that engaging with systems could have broader consequences has an effect both on how people perceive the risks and benefits of interacting with healthcare services or with any related system of complaints, advocacy or quality improvement.

### Expectations and experiences of submitting complaints or asking for help (‘calling the abyss’)

Participants described multiple barriers to reporting issues in healthcare services received (or not received) and highlighted that people in custody “can speak out for themselves, but who’s gonna listen?” (HG). The idea that it is difficult to make yourself heard in custody was reflected in perspectives on existing processes meant to advocate for or support people in custody such as the Ombudsman (an independent office that investigates and works to resolve complaints against government agencies and public services). Many participants had not heard of the Ombudsman or were unaware of the role of the office. Others described calling multiple times without getting through which one participant described as ‘calling the abyss’ (ST).

*Yeah, you can file. You absolutely have rights, like you can do this. But again, I don’t want to sound pessimistic, it’s - you’re writing a letter to yourself. It’s not going anywhere (BN).*

People also described the fear of repercussions for complaining or advocating for themselves. This was highlighted by multiple participants as a core challenge to people in custody being able to speak up about their healthcare.

*Once they know that you want to speak to the Ombudsman. They make your life harder in there (HG).*

Compounding challenges, these same systems may rely on staff to facilitate access to processes, such as submitting written requests for assistance, which can stop people from participating in the process all together. This was especially complex in situations where people were attempting to voice concerns that would affect (or were about) staff but relied on those same staff to be able to access the process.

*Like that [form] might not even make it off the range. Huh! Right into the garbage can like ‘boop!’ (NQ)*

In addition to physically preventing people from participating in these processes, these barriers acted as a self-censoring deterrent since people did not believe that they would be able to access the system and even if they did, it would not have an effect.

*What a waste of time, so you just stop. You won’t even engage anymore. I’ve seen that happen, where people come in like big advocates, and it dies like the quickest fire (BN).*

The intersecting barriers to engaging in current systems of complaints or requests for intervention affected how participants saw the potential efficacy of a patient feedback process as well as how likely people in custody would be to trust and engage in it.

### Key recommendations for a feedback process

**Figure 2.**
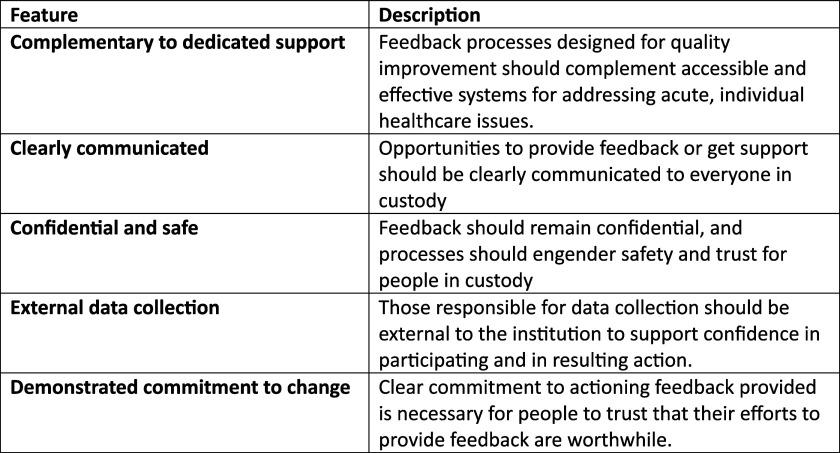
Recommended key features of processes to gather feedback for quality improvement of healthcare services delivered in custody.

We asked participants to imagine real opportunities for people in custody to provide feedback on their experiences of healthcare to inform policy, programming and quality improvement work. Given the challenges identified, there were no easy or obvious paths, but participants identified key features that should be included in developing feedback process including complementing dedicated strategies to address acute needs and issues, clear communication of opportunities to provide feedback, ensuring confidentiality and safety, data collection by individuals and organizations external to the institution, and demonstrating a commitment to change (Figure 2).

## Discussion

This study aimed to understand opportunities and barriers for people in correctional facilities to provide feedback on their experiences of healthcare services in custody. People with recent experience of incarceration identified multiple intersecting challenges to this type of work, centred on experiences and expectations of healthcare in custody and of existing processes for complaints and advocacy.

Participants in this study highlighted again and again that healthcare services were in many ways defined by the context of custody, and that the system was not designed for health or healthcare. The tension between care and security, and the effects on access and experiences of healthcare described by participants in this study have been identified in correctional contexts in other jurisdictions (Condon *et al*., 2007; Plugge *et al*., 2008; Nyvoll, 2025). Many of the specific impacts have been reported in other jurisdictions including challenges with access to medications (Condon *et al.*, 2007; Plugge *et al*., 2008; Nyvoll, 2025), lack of health autonomy (Nyvoll, 2025), ‘gatekeeping’ of access to healthcare services by non-healthcare, and healthcare staff (Condon *et al*., 2007; Plugge *et al.*, 2008), feeling that healthcare needs were not viewed as legitimate (Condon *et al*., 2007; Plugge *et al*., 2008; Nyvoll, 2025), and lack of confidentiality (Condon *et al*., 2007; Plugge *et al*., 2008).

In addition to shaping expectations of care, the environment of correctional facilities shaped perceptions of opportunities and barriers to providing feedback on healthcare services.

Participants in this study described fear of retaliation for voicing negative feedback. This was echoed in a study of two English prisons in which people in custody described fear of being identified as a barrier to participating in feedback mechanisms (Hankins *et al.*, 2022b). In their recommendations, participants in this study highlighted the need to ensure confidentiality and safety as a fundamental component of a feedback process. Our study also found feelings of powerlessness as underpinning experiences with healthcare or feedback mechanisms. In the same English study, some people reported providing feedback felt pointless and that complaints would not really be resolved (Hankins *et al*., 2022b). In a study of patient experiences of healthcare in custody in Norway, people described feeling that they ‘lacked the energy’ to fight for (or with) healthcare (Nyvoll, 2025). This is consistent with the recommendation that processes for feedback must demonstrate a clear commitment to change.

Our study has several strengths and limitations. Participants had diverse characteristics and experiences of incarceration. This broad perspective was a strength in identifying commonalities. We sought people with lived expertise of the provincial correctional system in a single province in Canada which may affect generalizability to other jurisdictions. However, similar findings with other studies in international contexts suggests common features of healthcare services delivered in custody and feedback processes.

In this study, people with recent experience of incarceration provided key insights into the barriers and opportunities for people in custody to providing feedback on healthcare services they receive. These insights may be applied across correctional settings to support meaningful engagement of services users in providing feedback. Future research and implementation science should explore the development of a structured patient experience feedback process in a custodial setting and evaluate how feedback from this process is integrated into improvements in policy and practice. Ultimately, ensuring that people receiving care in correctional facilities have a voice in improving that care is essential to improving quality of services and health outcomes for people who experience incarceration.

## Supporting information

10.1017/S1463423626101340.sm001 McLeod et al. supplementary material McLeod et al. supplementary material

## Data Availability

Supporting data are not available due to the sensitive nature of the research and to protect participant identities and confidentiality.

## Supplementary material

The supplementary material for this article can be found at https://doi.org/10.1017/S1463423626101340.
