# Supplementary material for: ‘But who’s gonna listen?’ A qualitative study of voicing experiences of healthcare in provincial correctional facilities in Ontario, Canada
Source: Prim Health Care Res Dev. 2026 Jun 29;27:e72. doi: 10.1017/S1463423626101340 (PMC13319478; doi:10.1017/S1463423626101340)
Supplement: McLeod et al. supplementary material [file S1463423626101340sup001.docx]

**APPENDIX A – Focus Group/Interview Guiding Questions**

1. What are the most important ways that healthcare in custody is different from healthcare in the community?
   1. Access to care
   2. Treatment and services
   3. Relationship between patients and providers
   4. Throughcare
2. What are the most important things that make healthcare good quality?
   1. Access to care?
   2. What are the most important qualities in your interactions with healthcare staff?
   3. What are the most important things to measure?
3. What are the ways that people in prisons can give feedback about the care they receive right now?
   1. How easy or difficult is it to give feedback?
   2. How does that information get used?
4. What do you think is the best way people in prisons could have a voice in improving healthcare?
   1. What are some of the challenges with collecting patient feedback?
5. Anything else we haven’t covered that is important to think about on this topic?
